# Attenuated Visual Function in Patients with Major Depressive Disorder

**DOI:** 10.3390/jcm9061951

**Published:** 2020-06-22

**Authors:** Kyoung In Jung, Seo-Yeon Hong, Da Young Shin, Na Young Lee, Tae-Suk Kim, Chan Kee Park

**Affiliations:** 1 Department of Ophthalmology, College of Medicine, The Catholic University of Korea, Seoul 06591, Korea; ezilean@hanmail.net (K.I.J.); lizhong3478@naver.com (S.-Y.H.); tlsek0@hanmail.net (D.Y.S.); nyny5555@naver.com (N.Y.L.); 2 Department of Ophthalmology, Seoul St. Mary’s Hospital, Seoul 06591, Korea; 3 Department of Ophthalmology, Eunpyung St. Mary’s Hospital, Seoul 03312, Korea; 4 Department of Psychiatry, Seoul St. Mary’s Hospital, College of Medicine, The Catholic University of Korea, Seoul 06591, Korea

**Keywords:** depression, neurodegeneration, optic nerve, retina, retinal ganglion cell, visual field

## Abstract

Background: We sought to investigate visual function, primarily, and structural changes in retinal ganglion cells, secondarily, in patients with major depressive disorder. Methods: A total of 50 normal participants and 49 patients with major depressive disorder were included in this cross-sectional study. The participants underwent 24–2 standard automated perimetry and spectral-domain optical coherence tomography. Results: The pattern standard deviation (PSD) in the visual field test was higher in the major depressive disorder patients than in the normal control subjects (*p* = 0.017). The patients with major depressive disorder showed reduced minimum ganglion cell–inner plexiform layer (GCIPL) thickness relative to the normal control participants (*p* = 0.015). The average score on the Hamilton Depression Rating scale showed a significant correlation with the PSD, minimum GCIPL thickness, and inferior GCIPL thickness (r = 0.265, *p* = 0.009; r = −0.239, *p* = 0.017; and r = −0.204, *p* = 0.043, respectively). The multivariate analysis of factors associated with PSD showed old age and a high Hamilton Depression Rating score to be relevant (*p* = 0.002 and 0.028, respectively). Conclusions: Visual function was decreased and the GCIPL thickness was reduced in major depressive disorder patients. The retinal neurodegenerative process in depression might be considered in patients with depression.

## 1. Introduction 

A growing body of literature suggests a higher prevalence of depressive symptoms can be found in patients with visual impairments, including glaucoma [1,2,3]. Decreased vision could lead to a low quality of life and could be followed by depressive symptoms [4,5]. In addition, the awareness of the disease itself or potential side effects of treatment could contribute to the development of anxiety and the fear of future visual loss because of glaucoma [3,6].

We previously reported that subjects with undiagnosed glaucoma were more depressed than those without glaucoma in a population-based study [7]. Without considering the perception of the disease or the side defects of treatment, glaucoma patients still seem to have a higher possibility of presenting with depressive symptoms.

Several studies have also found that depression is associated with the degree of decreased subjective visual function, such as difficulty with vision-related tasks, but not with a worse visual acuity or visual field (VF) test score [3,6]. That suggests that how subjects perceive their vision might be more significantly related to depressive symptoms than visual acuity or visual sensitivity as measured by the VF test [6]. In other words, the individual’s perception of worse vision might lead to depressive symptoms [6]. It also may be possible that depression results in decreased visual function, although no study so far has explored a cause-and-effect relationship [6].

Accumulating clinical evidence has supported the possibility of depression affecting retinal ganglion cells or glaucoma. Neuroimaging studies have postulated that depression could be a neurodegenerative process in key brain regions [8]. Patients with depression showed a decrease in the sizes, synapses, and density of neurons in the brain cortex [9,10]. Retinal ganglion cells may be affected by neurodegeneration in the brain because retrograde axonal transport is critical for the survival or maintenance of retinal ganglion cells [11,12]. The common risk factors for glaucoma and depression could link the two conditions. There is a wealth of evidence reporting that depression is associated with an increased risk of cardiovascular disease or autonomic dysfunction [13], and these are also known to be risk factors for glaucoma [14,15,16,17].

Several studies support the association between decreased volumes of the retinal ganglion cell layer and the inner plexiform layer and the thickness of the retinal nerve fiber layer (RNFL) with depression, although one study conversely reported that no such relationship existed [18,19,20]. To date, visual function using VF tests has not yet been extensively studied in patients with major depression, representing a gap in the literature.

In this study, we investigated visual function using VF tests as well as retinal ganglion cell-related structural parameters such as the retinal RNFL thickness, optic disc parameters, and ganglion cell–inner plexiform layer (GCIPL) thickness in patients with major depression.

## 2. Experimental Section

This study included patients with major depressive disorder, who were diagnosed based on the Diagnostic and Statistical Manual (DSM) of Mental Disorders, fifth edition criteria in the Department of Psychiatry at Seoul St. Mary’s Hospital, between September 2016 and April 2017. The control subjects were recruited by presenting advertising posters in our hospital. The Institutional Review Board of the Catholic University of Korea, Seoul, Korea approved this cross-sectional study. This study followed the tenets of the Declaration of Helsinki, and informed consent was obtained from all the participants prior to their inclusion.

Patients with comorbid first axis mental disorder diagnoses, severe immunological disease, or cerebrovascular disease were excluded. Furthermore, individuals with retinal disease or glaucoma, uveitis, ocular trauma, or other optic neuropathies were excluded. Finally, those with a best-corrected visual acuity of less than 20/40, axial length of more than 27 mm, or intraocular pressure of more than 21 mmHg were also excluded.

The Perceived Stress Scale (PSS)-10 is a self-reported questionnaire adopted to evaluate the degree of stress, specifically estimating the degree to which subjects consider their life has been unpredictable, uncontrollable, and overloaded during the previous month [21]. The Hamilton Depression Rating Scale (HDRS) was selected to assess the severity of depression in this study. The HDRS has been one of the most widely used depression severity rating scales and provides ratings of depression severity based on the current DSM-IV symptoms of depression [22]. To evaluate the confounding factors associated with depression, marital status, education level, smoking status, and occupational status were analyzed. Patients with “high education” meant those who were, at minimum, high school graduates.

All the subjects underwent several ophthalmic examinations, including best-corrected visual acuity, slit-lamp biomicroscopy, tonometry, axial length measurement (IOLMaster; Carl Zeiss Meditec, Dublin, CA, USA), and fundus photography. The averages of these ocular parameters from both eyes were analyzed.

All the patients underwent 24–2 standard automated perimetry with a Humphrey field analyzer (Carl Zeiss Meditec, Jena, Germany). The mean deviation and pattern standard deviation (PSD) were analyzed. Reliable tests were defined as those having less than 15% fixation losses, false positives, or false negatives. A second VF test was completed if the results of the first one were not reliable. If the results of the second test were similarly not reliable, the patient was excluded from further analysis.

Spectral-domain optical coherence tomography (OCT) imaging was performed using Cirrus HD-OCT version 6.0 (Carl Zeiss Meditec, Jena, Germany). Using a macular cube scan, the GCIPL thickness was obtained. Ganglion-cell analysis software was used to measure the average, minimum, and sectoral (i.e., superior, superotemporal, superonasal, inferior, inferotemporal, and inferonasal) GCIPL thickness parameters in a 14.13 mm^2^ elliptical annulus with vertical inner and outer radii of 0.5 and 2.0 mm, respectively, and horizontal inner and outer radii of 0.6 and 2.4 mm, respectively. The RNFL thickness was determined using the optic disc cube 200 × 200 scan mode. The average RNFL thickness or the mean thickness in each of the four quadrants provided by the software were analyzed. The optic disc parameters such as disc area, rim area, average cup-to-disc ratio (CDR), vertical CDR, and cup volume were assessed. The poor-quality images with a signal strength of less than 6 were discarded.

Statistical analysis was completed using the Statistical Package for the Social Sciences version 22.0 software program (IBM Corp., Armonk, NY, USA). For ocular variables such as visual fields and imaging, data from both eyes were averaged. The differences between the control subjects and patients with depression were assessed by the Student’s t-test or chi-squared test. The correlations between the parameters were evaluated according to Pearson’s correlation coefficient. A multivariate regression analysis of the factors associated with the VF parameters was performed. In the computation of correlations between the variables, correction was not carried out for multiple testing because this study was an explorative data analysis, as well in order to minimize the risk of type II errors. *p* < 0.05 indicated statistical significance.

## 3. Results

### 3.1. Baseline Characteristics

Among the recruited patients with depressive disorder, four patients were excluded because of the presence of retinoschisis (one patient), glaucoma (one patient), and the low reliability of the VF tests (two patients). Thus, the 49 patients with major depressive disorder and 50 control subjects were enrolled in this study. There was no significant difference in terms of age, gender, presence of diabetes or hypertension, marital status, or education level between the normal control subjects and patients with major depressive disorder (*p* > 0.05) (Table 1). The ophthalmologic examinations showed similar results with respect to axial length and intraocular pressure between the two groups (*p* > 0.05). The ratio of employment was lower among the patients with depression than that among the control subjects (*p* < 0.001).

### 3.2. Stress and Depression Scale

**Figure 1:**
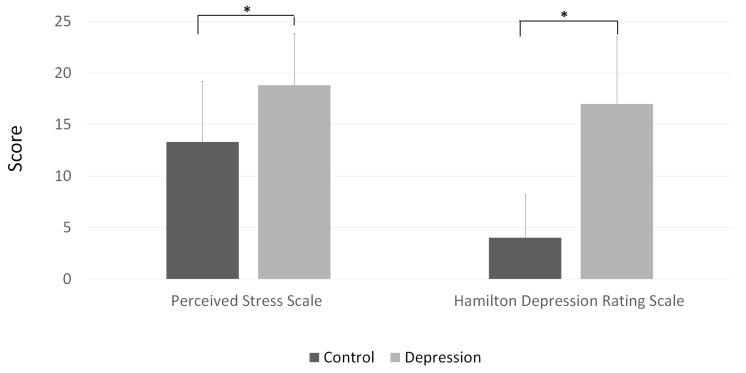
Perceived Stress Scale (PSS)-10 and Hamilton Depression Rating Scale (HDRS). A higher total score on the PSS-10 was recorded in the depression group than in the control group (*p* < 0.001). Patients with major depressive disorder also presented higher HDRS scores than the control subjects (*p* < 0.001).

The total stress scale score as discerned by the PSS-10 was 18.8 ± 5.0 in the depression group and 13.3 ± 5.9 in the control group (*p* < 0.001) (Figure 1). The HDRS score was also higher among the patients with major depressive disorder (17.0 ± 6.5) than among the control subjects (4.0 ± 4.2; *p* < 0.001).

### 3.3. Visual Field Test and Optical Coherence Tomography Parameters

For the VF test, the mean deviation appeared to be lower while the PSD was higher in the major depression group than in the control group, but statistical significance was present only for the comparison of the PSD (*p* = 0.017) (Table 2).

Considering the optic disc parameters, there was no significant difference between the control group and the depression group (*p* > 0.005). The average or individual quadrant parapapillary RNFL thickness value was thinner in the patients with major depression than in the control group, but there was no significant difference between the two groups (all *p* > 0.05). The minimum GCIPL thickness was significantly thinner in patients with major depressive disorder (77.5 ± 7.0 μm) relative to that among the control subjects (80.4 ± 4.4 μm; *p* = 0.015).

### 3.4. Relationship Between Visual Field Test Parameters or Optical Coherence Tomography and Stress or Depression Scale

**Figure 2:**
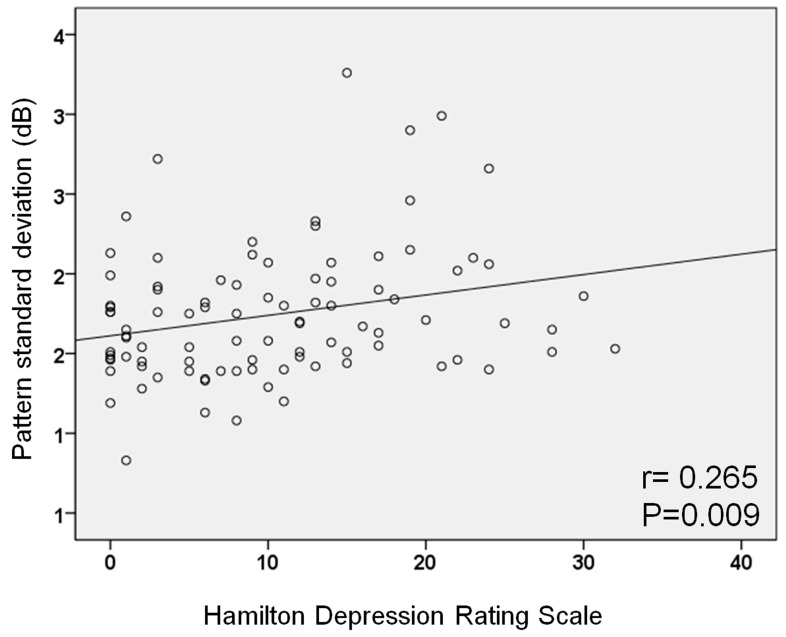
Correlations between Hamilton Depression Rating Scale (HDRS) score and pattern standard deviation (PSD) on visual field test. The severity of depression as measured by the HDRS was positively correlated with PSD.

The depression severity was also positively associated with the PSD (r = 0.265; *p* = 0.009) (Figure 2). The total stress scale score using the PSS-10 negatively correlated with the inferior RNFL thickness and average, inferonasal, inferior, and inferotemporal GCIPL thickness (all *p* < 0.05) (Supplemental Table S1). The depression severity as measured by the HDRS was significantly related to the minimum GCIPL thickness (r = −0.239; *p* = 0.017) and inferior GCIPL thickness (r = −0.204; *p* = 0.043; Supplemental Table S1).

The multivariate analysis of factors associated with the PSD on VF tests revealed older age (*p* = 0.002) and the severity of depression as measured by the HDRS to be relevant (*p* = 0.028) (Table 3).

### 3.5. Representative Cases

**Figure 3:**
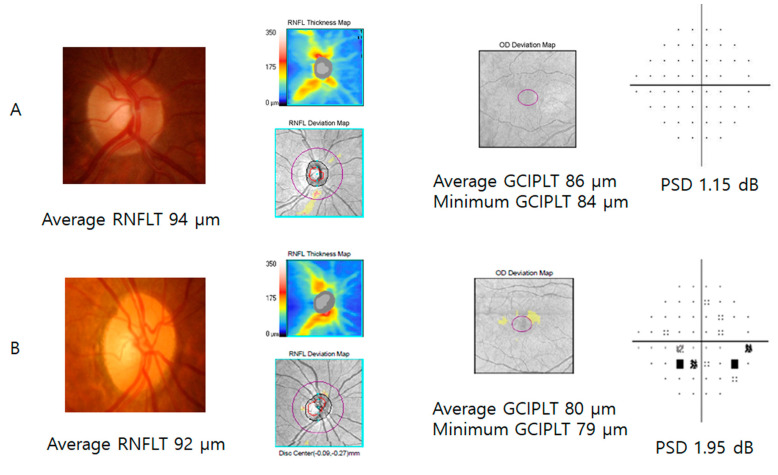
A sixty-three-year-old female with major depression (**B**) presented lower ganglion cell–inner plexiform layer thickness (GCIPLT) than a sixty-five-year-old female without depression (**A**). The patient with depression showed a higher pattern standard deviation (PSD = 1.95 dB) than the subject without it (PSD = 1.15 dB).

Representative cases are presented in Figure 3. A 63-year-old female with major depression presented thinner GCIPLs than a 65-year-old female without depression. The patient with depression also demonstrated depressed retinal sensitivity in the VF test. The PSD was higher in the patient with depression (1.95 dB) than in the subject without it (1.15 dB).

### 3.6. The Influence of Drug Use by Patients with Major Depressive Disorder on GCIPL Thickness

In the subgroup analysis to evaluate the influence of drug use by the patients with major depressive disorder, the patients taking selective serotonin reuptake inhibitors (SSRIs) showed thicker average GCIPLs as compared with those not taking these medications (*p* = 0.033) (Supplementary Table S2).

## 4. Discussion

We determined that the patients with depression displayed reduced visual function and thinner minimum GCIPLs relative to the control subjects. The more severe the depression was, the lower the visual function and the thinner the GCIPL. Older age and the severity of depression were factors found to be associated with VF abnormality in the multivariate analysis.

Among the patients with depression, the PSD with regard to visual function was higher than in the normal control subjects. During multivariate analysis, the severity of depression as measured by the HDRS was significantly associated with the PSD (*p* = 0.028). A high PSD indicates a nonuniform visual sensitivity loss. In glaucoma, the PSD has been found to be a critical index for detecting early glaucomatous damage in several studies, including the Ocular Hypertension Treatment Study [23,24]. The PSD provides information about focal VF loss, which is common in the early stages of the diseases [24,25]. Aside from the VF testing, reports using electrophysiologic examinations suggested that retinal physiology might be affected in patients with major depressive disorder. A reduction in the contrast gain of pattern electroretinograms in patients with major depressive disorder has been reported by several studies, even though one study found no difference in this regard between patients with depression and control subjects [26,27,28,29]. One explanation here could be that the different level of neurotransmitter activities in patients with depression might lead to a decreased retinal response [30]. Alternatively, social and physical anhedonia, which may be found in major depression, could be linked to a decreased response to sensory–visual input with changing levels of luminance contrast [31]. Therefore, we evaluated the structural parameters of retinal ganglion cells able to exclude the effects of anhedonia concurrently with conducting VF testing. Decreased GCIPL thickness was verified in the patients with depression in this study.

In this study, we found no difference in the optic disc parameters such as the rim area or CDR between the patients with depression and the control subjects. To our knowledge, no study has yet evaluated the optic disc parameters in patients with major depression. There was also no significant difference found in the parapapillary RNFL thickness between the depression group and the control group, corresponding to the outcomes of Sönmez et al.’s study [20].

On the other hand, the minimum GCIPL thickness presented a significant distinction between the patients with major depressive disorder and the control subjects. The GCIPL thickness reflected somas of retinal ganglion cells from the ganglion cell layer and dendrites of retinal ganglion cells or synapses between retinal ganglion cells and bipolar or amacrine cells from the inner plexiform layers of the retina [32,33]. Upon OCT examination, the GCIPL thickness was evaluated mostly in the macular area of the retina, where a high proportion of retinal ganglion cells are located. The discovery of the association between depression and a thinner GCIPL supports Kalenderoglu et al.’s OCT findings of decreased ganglion cell layer and inner plexiform layer volumes in patients with major depressive disorder [18]. We also found correlations between the minimum or inferior GCIPL thickness and the severity of depression. Elsewhere, Yildiz et al. observed no difference in GCIPL thickness between the major depression patients and normal control subjects but at least noted a correlation between GCIPL and the duration of the latest depressive episode [19].

In this study, a significant difference between the depressive patients and the controls was found only in terms of the GCIPL thickness rather than also pertaining to the parapapillary RNFL or optic disc parameters. Changes in certain optic disc parameters, such as an increased CDR or rim change accompanying decreased parapapillary RNFL thickness, are pathognomonic features of glaucoma, even in the early stages [34]. Given the impact of these features in this study, the reduced GCIPL thickness in patients with depression might be induced by a different mechanism from that of glaucoma.

Several assumptions could potentially explain the finding of thinner GCIPLs among depressed patients. Firstly, a neurodegenerative process has also been suggested in the pathophysiology of major depression [35]. Volumetric MRI research revealed reductions in grey matter affecting neuronal cell bodies, glial cells, and synapses in patients with depression [36]. Postmortem research reported findings of reduced synapse numbers and neuronal density in the prefrontal cortices of patients with depression [10]. It has been known that the molecular pathway in major depressive disorder combines dopamine and serotonin, which are involved in retinal processes [30]. Therefore, it might be possible that the dendrites of retinal ganglion cells or synapses between the retinal ganglion cells or other retinal cells in the inner plexiform layer are altered in patients with depression.

Secondly, stress could reduce the level of brain-derived neurotrophic factor in major depressive patients [37]. The axons of retinal ganglion cells project to the lateral geniculate body, which conveys visual information to the primary visual cortex. Retrograde or antegrade axonal transport is critical for the survival and maintenance of retinal ganglion cells [11,12]. The dendrites and somas of retinal ganglion cells in the retina might be disrupted as the process of transneuronal retrograde degeneration in patients with depression progresses. However, this speculation requires further investigation for confirmation.

In the subgroup analysis of drugs used in patients with major depressive disorder, using SSRIs was associated with thicker GCIPLs. Disturbed serotonin function could play a role in the pathophysiology of depression [38]. Serotonin is one of the neurotransmitters detected in the mammalian retina, and serotonin receptors have been found in bipolar cells [39]. The relationship between using SSRIs and thicker GCIPL thickness corresponds to one meta-analysis suggesting a relationship between the use of SSRIs and a lower unadjusted risk of the development of primary open-angle glaucoma, even though the correlation was nonsignificant after adjustment for confounding factors [40]. Kalenderoglu et al. also reported no relationship between the use of SSRIs and retina OCT parameters [18]. In this study, there was no significant relationship between the use of SSRIs and VF parameters. Further well-controlled, longitudinal studies are needed to establish the nature of the relationship between SSRIs and retinal neurodegeneration.

One of the limitations of our study is that most of the patients with depression were on drugs, with the exception of four patients. Previously, potential ocular complications of antidepressants have been reported [41,42]. We evaluated the effects of drugs received by patients on OCT parameters. However, retinal degenerative changes below the diagnostic threshold could not be completely excluded, even though individuals with retinal disease were not included in this study. This study was a cross-sectional study, so causation cannot be established. Further prospective longitudinal research is needed to reveal the relationship between depression and the risk of retinal neuronal degeneration. Previous studies evaluating OCT parameters in patients with depression did not include the data on axial length without mention of the exclusion of highly myopic eyes [18,19]. The strength of our study was that we measured axial length and then excluded highly myopic eyes with an axial length of more than 27 mm, because a very long axial length could affect the measurements on OCT parameters.

## 5. Conclusions

In this study, we demonstrated that depressive patients without ophthalmic diseases had reduced visual function and thinner retinal thickness parameters (GCIPLs). The more severe the depression, the more attenuated the visual function and the thinner the GICPLs. Given these findings, there is a need to consider that neurodegenerative process might occur in retinal ganglion cells or other retinal cells in depressive patients, although further investigation is required on this front to elucidate the exact mechanism and the causal relationship.

## Figures and Tables

**Table 1:** Demographics.

Parameters		Control (n = 50)	Depression (n = 49)	*p* Value
**Age**		45.5 ± 13.2	48.1 ± 15.7	0.377
**Gender (male/female)**		15/35	15/34	0.947
**Diabetes mellitus**		1 (2.0%)	4 (8.0%)	0.204
**Systemic hypertension**		6 (12.0%)	6 (12.0%)	1.000
**Ophthalmologic variables**	**Spherical equivalent (diopter)**	−1.2 ± 2.3	−1.5 ± 2.8	0.554
	**Axial length (mm)**	24.3 ± 1.2	24.0 ± 1.5	0.344
	**Intraocular pressure (mmHg)**	14.0 ± 2.6	14.1 ± 2.4	0.873
**Socio-economic variables**	**Marital status (marriage)**	64.6%	73.7%	0.366
	**Education level (high)**	91.7%	81.6%	0.203
	**Current smoker (%)**	10.4%	21.1%	0.229
	**Employment (%)**	72.9%	31.6%	<0.001 *

* Statistically significant difference between the groups, with *p* < 0.05.

**Table 2:** Visual field parameters, parapapillary retinal nerve fiber layer thickness, and ganglion cell–inner plexiform layer thickness in subjects with and without depression.

OCT and Visual Field Parameter		Control	Depression	*p* Value ^a^
**Visual field, dB**	**Mean deviation**	−1.9 ± 1.8	−2.4 ± 2.0	0.177
	**Pattern standard deviation**	1.6 ± 0.3	1.8 ± 0.5	0.017 *
**Optic disc parameter**	**Disc area (mm^2^)**	1.3 ± 0.2	1.3 ± 0.2	0.836
	**Rim area (mm^2^)**	1.9 ± 0.4	1.9 ± 0.3	0.905
	**Average CDR**	0.5 ± 0.2	0.5 ± 0.2	0.960
	**Vertical CDR**	0.5 ± 0.2	0.5 ± 0.2	0.716
	**Cup volume (mm^3^)**	0.2 ± 0.1	0.2 ± 0.1	0.714
**Parapapillary RNFL thickness,** μm	**Average**	96.6 ± 7.7	93.8 ± 7.8	0.077
	**Superior**	121.1 ± 13.6	118.2 ± 13.2	0.278
	**Nasal**	66.7 ± 7.4	64.7 ± 7.2	0.190
	**Inferior**	124.3 ± 14.5	121.2 ± 15.6	0.306
	**Temporal**	74.1 ± 13.4	71.2 ± 12.6	0.271
**GCIPL thickness,** μm	**Average**	83.1 ± 4.4	81.6 ± 6.2	0.154
	**Minimum**	80.4 ± 4.4	77.5 ± 7.0	0.015 *
	**Superior**	84.3 ± 5.0	82.8 ± 7.0	0.225
	**Superonasal**	85.3 ± 4.9	84.3 ± 6.1	0.409
	**Inferonasal**	83.5 ± 5.1	81.8 ± 5.9	0.127
	**Inferior**	81.0 ± 4.6	78.7 ± 7.0	0.058
	**Inferotemporal**	83.1 ± 5.3	81.3 ± 6.8	0.141
	**Superotemporal**	82.0 ± 4.8	80.6 ± 6.9	0.233

CDR, cup-to-disc ratio; GCIPL, Ganglion cell–inner plexiform layer; OCT; optical coherence tomography; RNFL, retinal nerve fiber layer. * Statistically significant difference between the groups, with *p* < 0.05.

**Table 3:** Multivariate analysis of factors associated with pattern standard deviation on visual field tests.

	Pattern Standard Deviation, dB		
	Beta	95% CI	*p* Value
Age (years)	0.010	0.004–0.016	0.002 *
Hamilton Depression Rating Scale	0.013	0.001–0.025	0.028 *
Employment	0.030	−0.161–0.221	0.754

* Statistically significant values (*p* < 0.05) are in bold.

## Supplementary Materials

The following are available online at https://www.mdpi.com/2077-0383/9/6/1951/s1. Table S1: Relationship between parapapillary retinal nerve fiber layer and ganglion cell-inner plexiform layer thickness, and visual field parameters with the Perceived Stress Scale (PSS)-10 or Hamilton Depression Rating Scale, Table S2: Peripapillary average retinal nerve fiber layer and ganglion cell–inner plexiform layer thickness, and visual field parameters according to different type of medication
