# Supplementary material for: Attenuated Visual Function in Patients with Major Depressive Disorder
Source: J Clin Med. 2020 Jun 22;9(6):1951. doi: 10.3390/jcm9061951 (PMC7356808; doi:10.3390/jcm9061951)
Supplement: Supplementary file 1 [file jcm-09-01951-s001.zip › Supplemntary files/Supplemental Table 1.pdf]

Supplemental table 1. Relationship between parapapillary retinal nerve fiber layer and ganglion cell-inner plexiform layer thickness, and visual field parameters with the Perceived Stress Scale (PSS)-10 or Hamilton Depression Rating Scale

		**PSS-10 (total)**		**Hamilton Depression Rating Scale(total)**	
		**r**	***P* value**	**r**	***P* value**
**RNFL thickness, μm**	Average	-0.174	0.085	-0.145	0.153
	Superior	-0.102	0.301	-0.093	0.362
	Nasal	-0.112	0.269	-0.158	0.118
	Inferior	**-0.228**	**0.023***	-0.108	0.289
	Temporal	0.025	0.804	-0.020	0.845
**GCIPL thickness, μm**	Average	**-0.215**	**0.033***	-0.135	0.183
	Minimum	-0.194	0.054	**-0.239**	**0.017***
	Superior	-0.192	0.058	-0.137	0.176
	Superonasal	-0.195	0.053	-0.094	0.352
	Inferonasal	**-0.230**	**0.022***	-0.115	0.256
	Inferior	**-0.267**	**0.008***	**-0.204**	**0.043***
	Inferotemporal	**-0.220**	**0.029***	-0.125	0.217
	Superotemporal	-0.128	0.208	-0.094	0.354
**Visual field**	MD	0.119	0.247	-0.073	0.481
	PSD	-0.060	0.558	**0.265**	**0.009***

GCIPL, ganglion cell-inner plexiform layer; MD, mean deviation; PSD, pattern standard deviation; RNFL, retinal nerve fiber layer

r=Pearson's correlation coefficient

*Statistically significant values ($P < 0.05$) are in bold.
