# Supplementary material for: Attenuated Visual Function in Patients with Major Depressive Disorder
Source: J Clin Med. 2020 Jun 22;9(6):1951. doi: 10.3390/jcm9061951 (PMC7356808; doi:10.3390/jcm9061951)
Supplement: Supplementary file 1 [file jcm-09-01951-s001.zip › Supplemntary files/Supplemental Table 2.pdf]

Supplemental table 2. Peripapillary average retinal nerve fiber layer and ganglion cell-inner plexiform layer thickness, and visual field parameters according to different type of medication

		Using SSRI		P value	Using SNRI		P value	Using antipsychotics		P value
		**No (n=17)**	**Yes (n=32)**		**No (n=40)**	**Yes (n=9)**		**No (n=40)**	**Yes (n=9)**	
**RNFL thickness,** μm	Average	92.5±7.9	94.6±7.8	0.378	93.5±7.3	95.4±10.3	0.501	93.2±7.2	96.6±10.3	0.252
**GCIPL thickness,** μm	Average	79.0±6.6	82.9±5.6	**0.033**	81.5±6.5	81.9±4.8	0.858	81.6±6.1	81.4±7.1	0.955
	Minimum	75.2±9.1	78.8±5.4	0.095	77.5±7.3	77.8±5.9	0.908	77.9±7.3	76.1±5.6	0.507
**Visual field**, dB	MD	-2.0±2.2	-2.5±1.9	0.427	-2.3±2.0	-2.7±1.9	0.639	-2.3±1.9	-2.6±2.1	0.698
	PSD	1.7±0.4	1.9±0.5	0.273	1.9±0.5	1.7±0.4	0.297	1.8±0.4	2.0±0.5	0.296

Selective serotonin reuptake inhibitor, SSRI
